# Supplementary material for: Gene‐specific correlation of RNA and protein levels in human cells and tissues
Source: Mol Syst Biol. 2016 Oct 26;12(10):883. doi: 10.15252/msb.20167144 (PMC5081484; doi:10.15252/msb.20167144)
Supplement: Supplementary file 1 — Expanded View Figures PDF [file MSB-12-883-s001.pdf]

Expanded View Figures

Figure EV1. Protein copy number and RNA levels (TPM) for all quantified genes.
The absolute copy numbers of proteins (blue) and the level of RNA (purple) measured as TPM are shown for all 55 genes across nine cell lines and 11 tissues. All values can be found in Table EV3 and Table EV6.

© 2016 The Authors

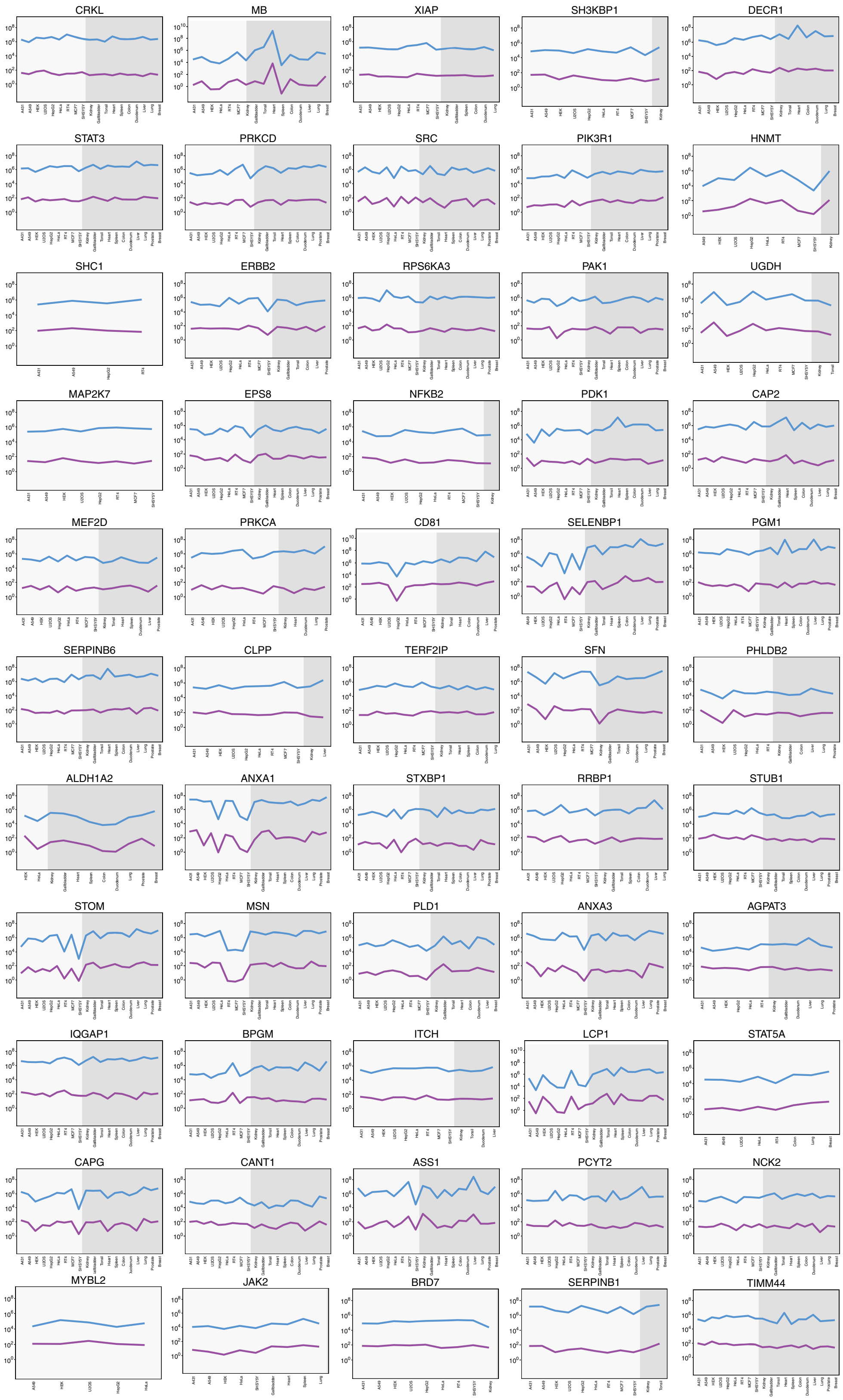

Figure EV1.

 © 2016 The Authors

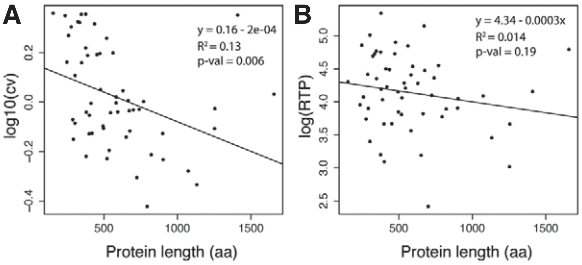

Figure EV2. RNA-to-protein ratio and variation versus protein length.

A The variation measured as coefficient of variation (cv) across samples are plotted against the protein length.

B The protein lengths for the 55 target proteins are plotted against the RTP ratio.

Data information: Test based on Pearson's product moment correlation coefficient and follows a *t*-distribution with length(x)-2 degrees of freedom if the samples follow independent normal distributions. An asymptotic confidence interval is given based on Fisher's *Z*-transform.

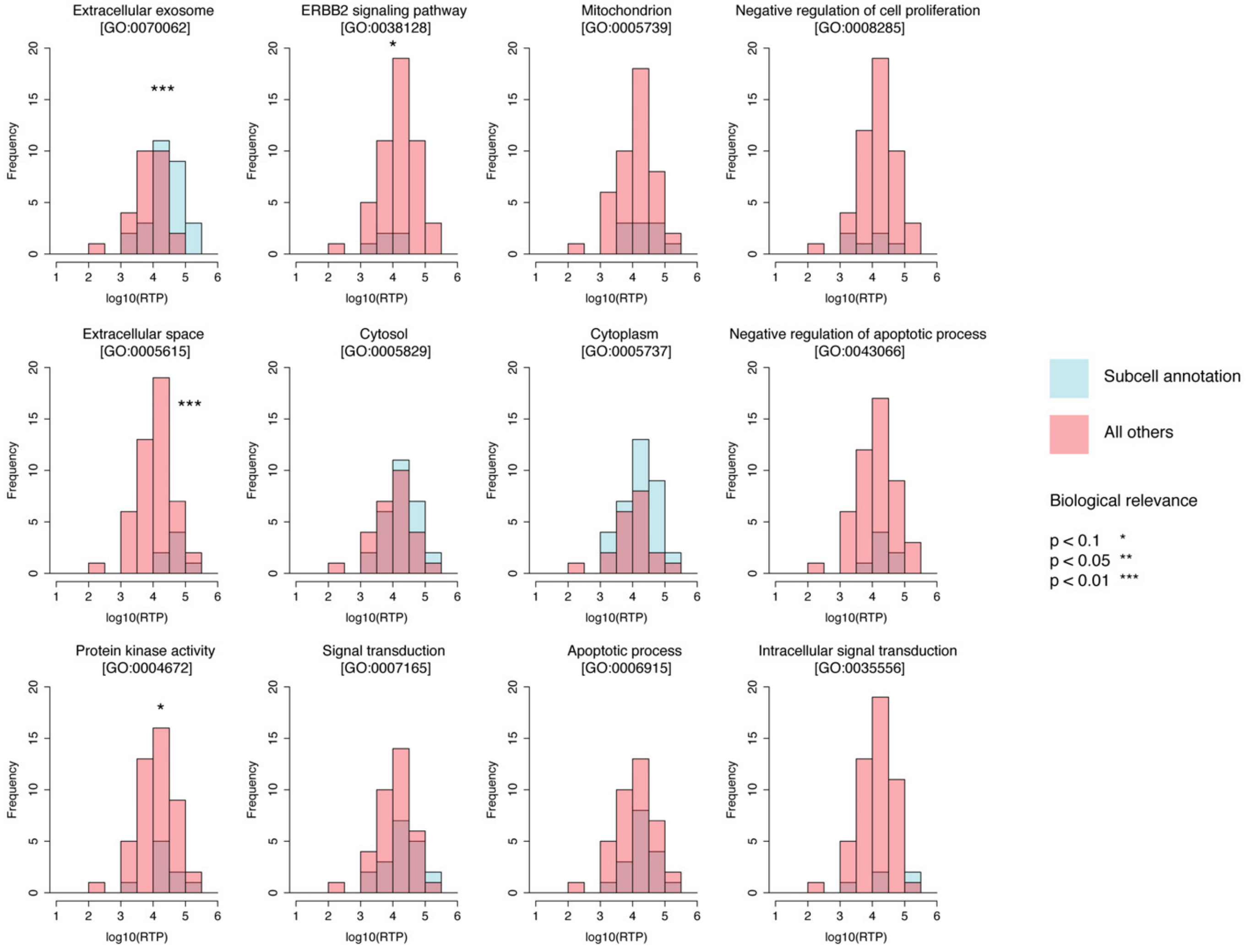

Figure EV3. RNA-to-protein ratios for proteins with different subcellular compartments.

The number of GO-annotated (UniProt, August 1, 2016) proteins in each compartment with a given RTP ratio is plotted (blue) with all other proteins not annotated to this compartment (red). *P*-values are calculated using Student's *t*-test.

© 2016 The Authors

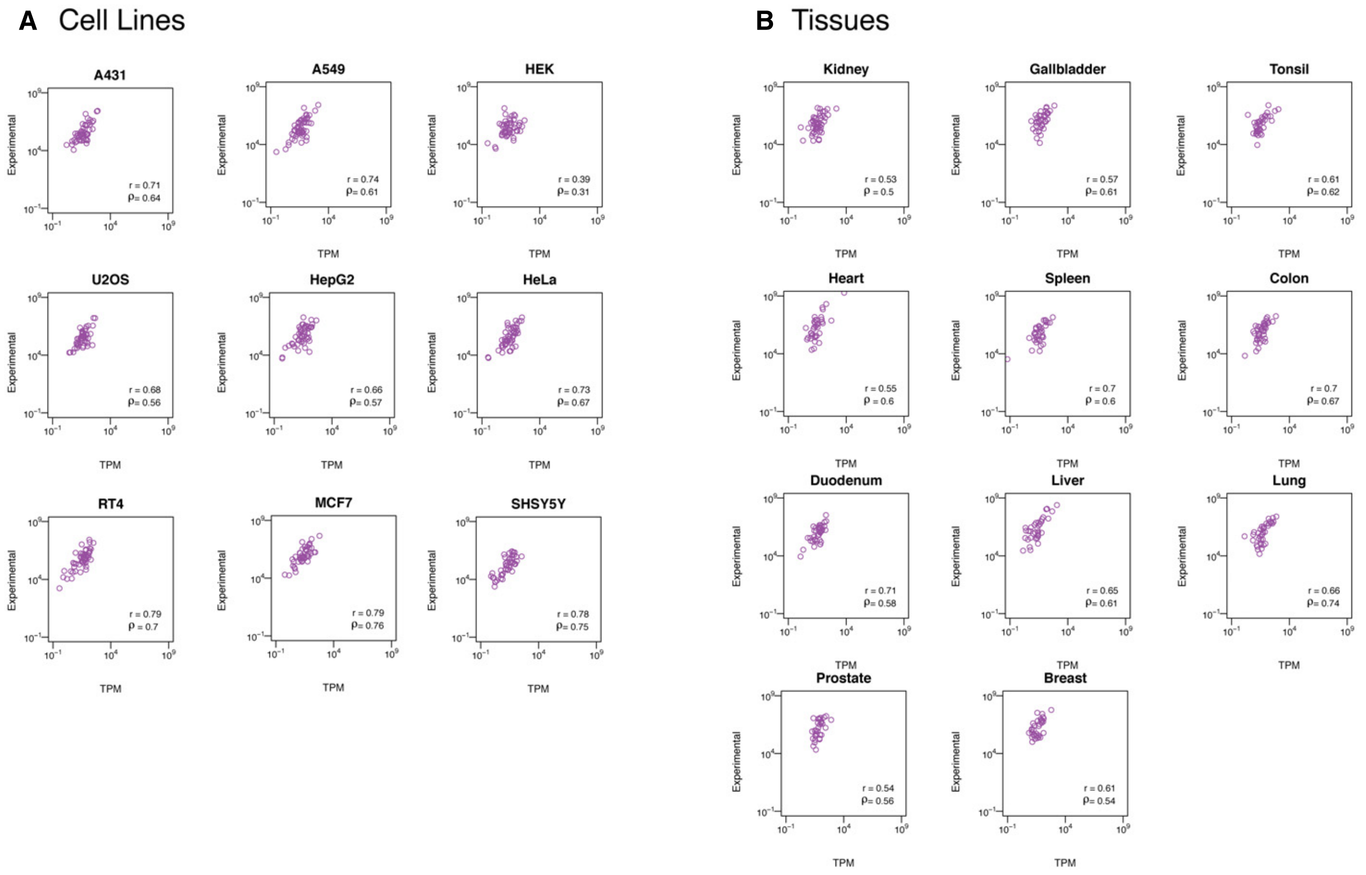

Figure EV4. Protein copies per cell versus RNA levels (TPM) for all quantified genes in all cell lines and tissues.

A, B The direct correlation between RNA (TPM, *y*-axis) and protein abundances (copy number, *x*-axis) for all quantified genes in all cell lines (A) and tissues (B). The Spearman's (ρ) and Pearson's (*r*) correlations between the two values across the quantified genes are shown.

 © 2016 The Authors

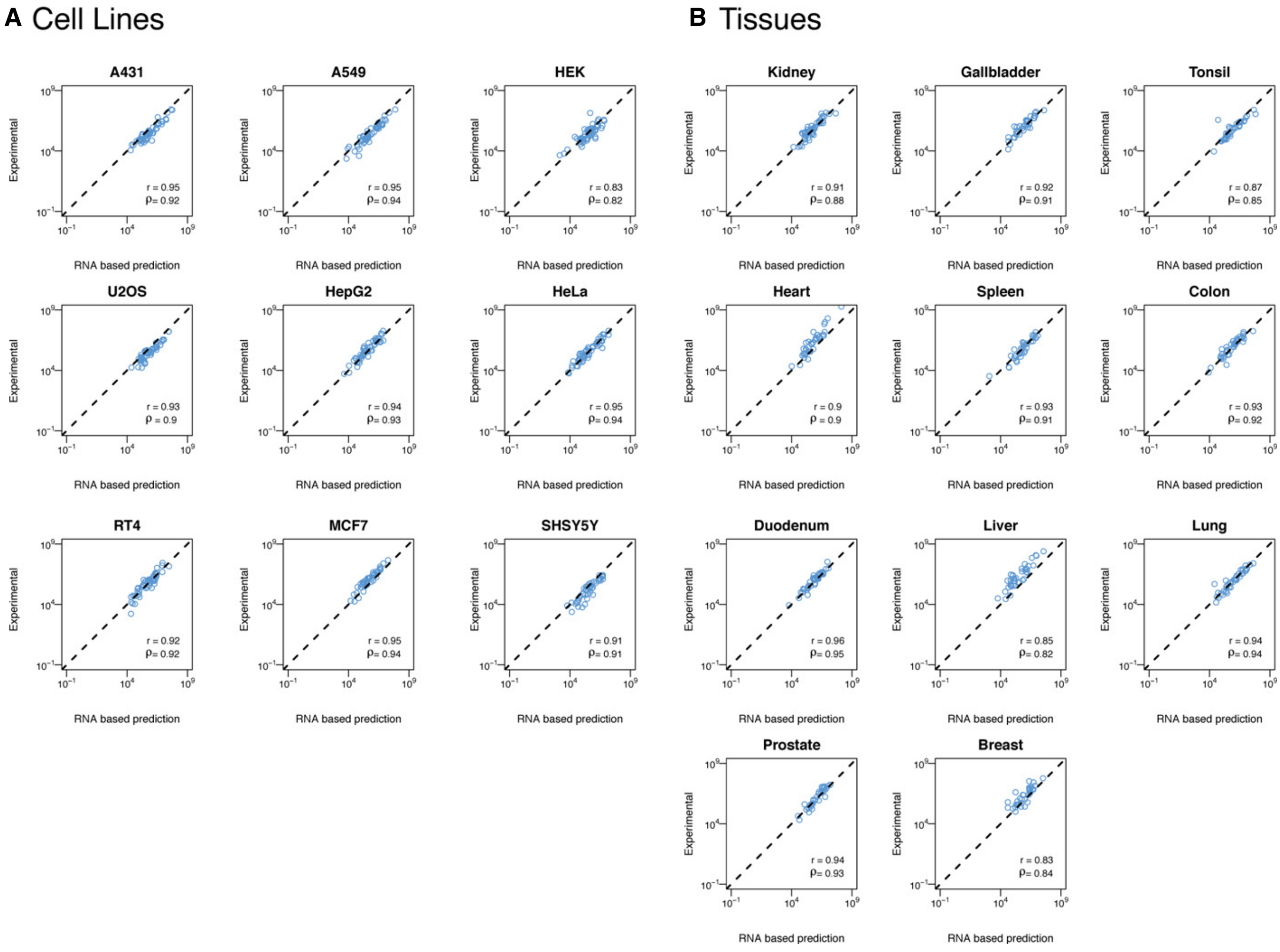

Figure EV5. Prediction of protein levels based on TPM levels as compared to the experimentally derived protein copy number for all quantified genes in all cell lines and tissues.

A, B The gene-specific correlation between protein copy number (*x*-axis) and predicted protein copy number based on the RNA levels (RNA-based prediction, *y*-axis) in all cell lines (A) and tissues (B). The Spearman's (ρ) and Pearson's (*r*) correlations between the two values across the quantified genes are shown.

© 2016 The Authors

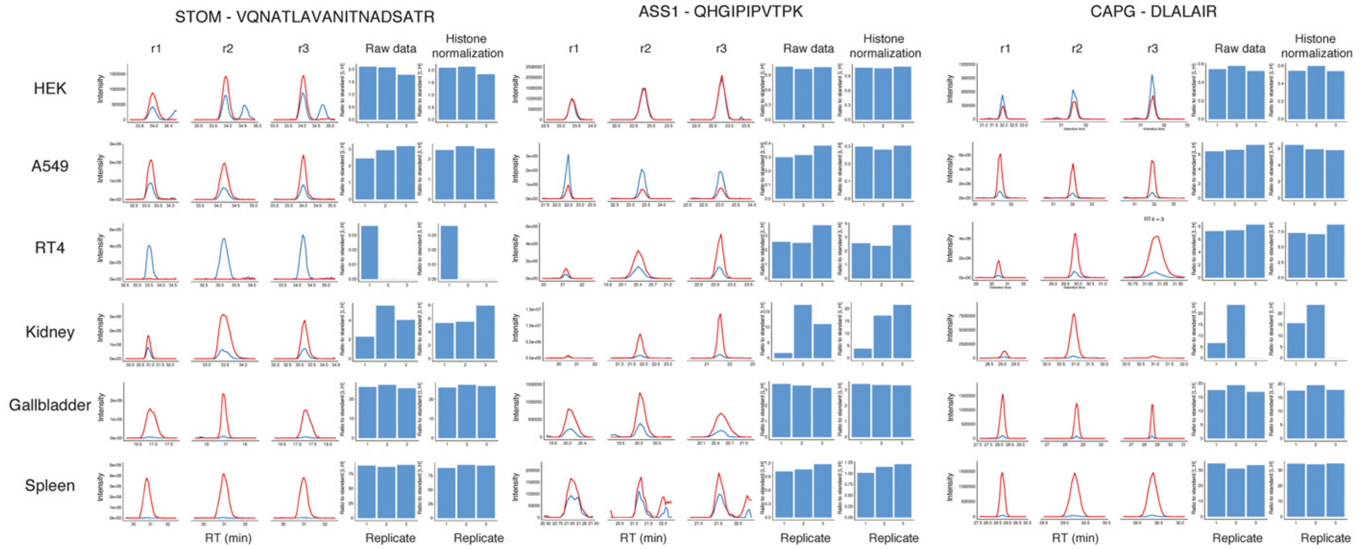

Figure EV6. Examples of quantitative peptide profiles using QPrEST for targeted proteomics.
All peptides were quantified by comparing the relative amount of light endogenous peptide (red) against the heavy standard peptide (blue). The relative amount was thereafter normalized against the number of cells present in each biological replicate by accounting for the relative amount of histones present in each sample.

 © 2016 The Authors
